# Delayed occurrence of foreign body reaction caused by poly‐L‐lactic acid with monitoring through ultrasonography

**DOI:** 10.1111/srt.13683

**Published:** 2024-04-02

**Authors:** Suk Bae Seo, Soo‐Bin Kim, Kyu‐Ho Yi

**Affiliations:** ^1^ SeoAhSong Dermatologic Clinic Seoul South Korea; ^2^ Division in Anatomy and Developmental Biology Department of Oral Biology Human Identification Research Institute, BK21 FOUR Project, Yonsei University College of Dentistry Seodaemun‐gu Seoul South Korea

Dear Editor,

Addressing age‐related changes and contour deformities has remained a longstanding concern within the medical community. Throughout time, a variety of materials have been employed as agents for tissue augmentation, administered for cosmetic purposes.^1^, ^2^ PLLA filler injections are frequently preferred for managing facial aging due to their stability and straightforward procedure. In 2004 as the first PLLA injectable facial volumizer for treating lipoatrophy, PLLA fillers have gained popularity for their efficacy and ease of use.

PLLA, is a gel polymer containing PLLA microspheres dispersed in water, accompanied by mannitol and carboxymethyl cellulose. Since its authorization, this injectable filler has been employed for medical applications, including addressing lipoatrophy in individuals with HIV, as well as for aesthetic improvements. Its function involves triggering a subtle inflammatory reaction, prompting the growth of fibroblasts and the synthesis of collagen, thereby resulting in a gradual augmentation of volume in dermal and subcutaneous layers.^3^

Delayed immune‐mediated adverse effects associated with PLLA may manifest years after administration, with reported onset ranging from 6 to 60 months.^4^, ^5^ These effects, including inflammatory nodules, papules, and swelling, can cause distress due to their resemblance to infectious conditions and potential cosmetic disfigurement. Notably, we present a case of granuloma formation occurring over 100 months post‐treatment, surpassing the previously reported maximum latency of 70 months for PLLA.^6^ This duration significantly exceeds the typical tissue longevity of the product, which is usually expected to last up to 2 years.^7^ This case underscores the importance for healthcare providers to recognize the full spectrum of adverse events related to procedural complications, ensuring informed patient consent and including this etiology in the differential diagnosis for patients presenting with facial nodules of uncertain origin, irrespective of the duration since exposure to PLLA.

*CASE*. A 52‐year‐old woman visited clinic with a non‐inflammatory nodule on her left nasolabial fold, which had been present for 1 month at the time of consultation. The nodule was firm and hardened, devoid of redness, pain, swelling, drainage, or ulceration. The patient was afebrile, and her overall medical history was unremarkable. She had previously undergone PLLA injections bilaterally in the nasolabial fold, using 1 vial, without any subsequent injections. Her exposure to PLLA (Sculptra, Bridgewater, NJ) began 9 years prior with a single injection of 7 mL of PLLA diluted in 9 mL of normal saline in the nasolabial fold. Physical examination revealed multiple well‐defined, ellipse‐shaped, firm, skin‐colored nodules on the left nasolabial fold (See Figure 1 and Video S1). Ultrasonographic assessment of the cheek depicted granulomas appearing as hyperechoic lesions beneath the dermal layer (See Figure 2).^8^

**FIGURE 1:**
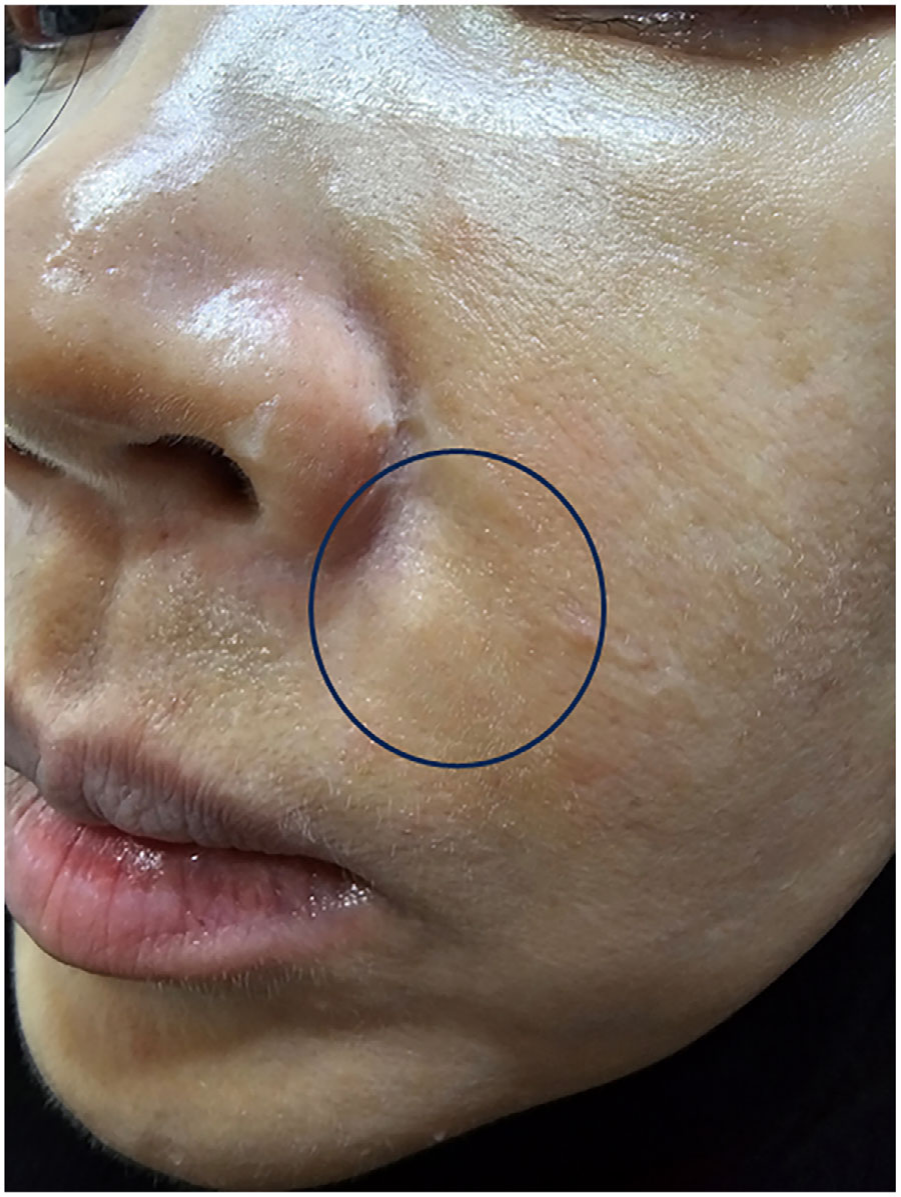
A 52‐year‐old woman without immune system deficiencies came in with a solitary, visible nodule on her left nasolabial fold, which had been there for one month when she sought medical attention. The nodule was solid and firm, showing no signs of redness, pain, swelling, discharge, or ulceration.

**FIGURE 2:**
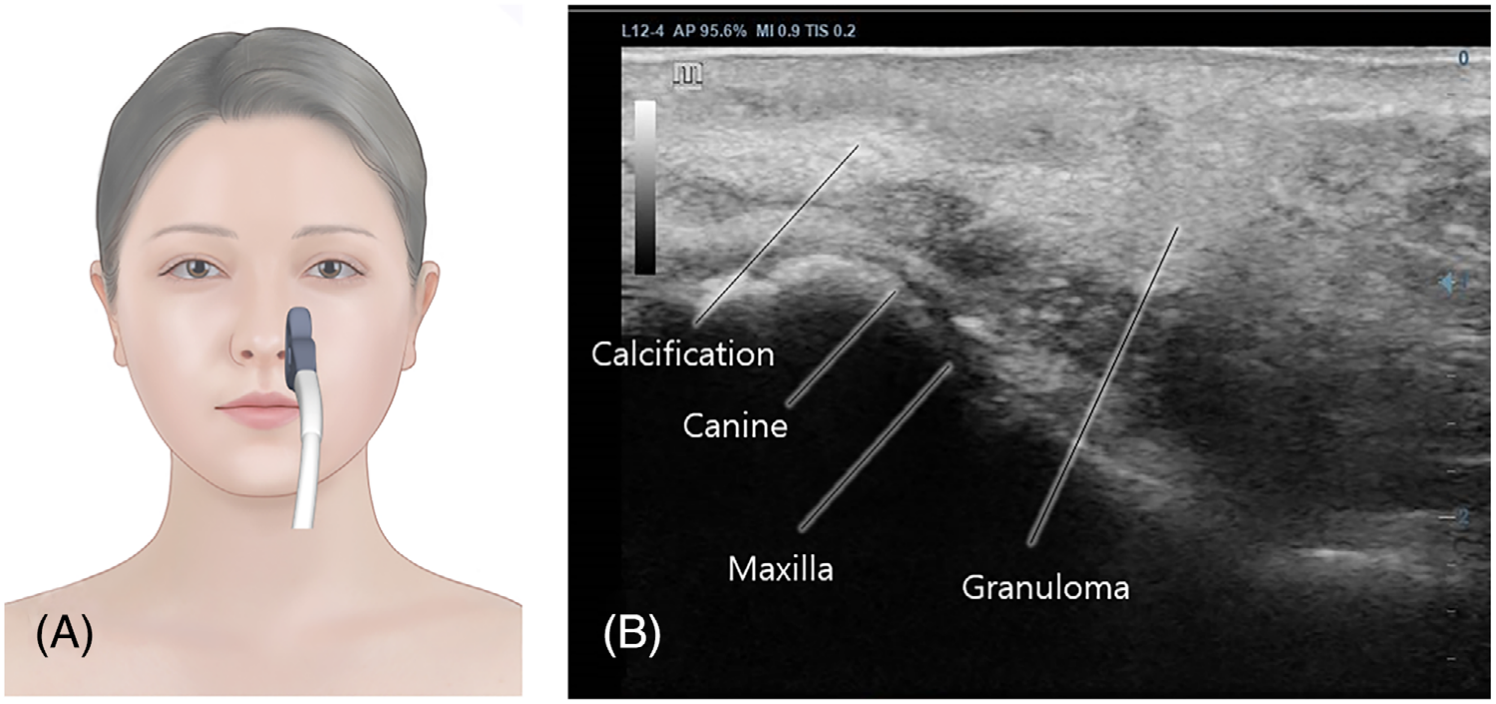
Ultrasonographic assessment of the left nasolabial fold in the longitudinal plane (A) depicted granulomas appearing as hyperechoic lesions beneath the dermal layer (B).

Granulomatous reactions to PLLA can manifest late after its approval for addressing lipoatrophy associated with aging and HIV antiretroviral therapy.^5^, ^7^ The formation of these granulomas might be linked to localized accumulation of PLLA, often due to insufficient post‐injection massage or incorrect injection depth. Although typically observed in mobile areas such as the nasolabial fold, the occurrence of a late‐onset granuloma in the temporal region, as evidenced in this case, prompts consideration of its aesthetic significance. While dilution with bacteriostatic water and lidocaine is generally regarded as adequate for preventing granuloma formation, variations in dilution levels could influence outcomes.^4^, ^5^, ^6^, ^9^ Histopathological examination confirmed the accurate subcutaneous injection depth; however, similarities with surgical sutures suggest the possibility of delayed granuloma formation, as observed in this instance. Although clinical resolution was achieved, the effectiveness of intralesional steroid therapy remains uncertain, underscoring the importance of cautious patient counseling regarding potential complications associated with PLLA injections.^10^

Yi et al.’s earlier study^8^ noted the development of granulomas following hyaluronic acid filler injections in the chin area. They found that these granulomas exhibited a hyperechoic appearance around the granuloma on ultrasonography.

PLLA remains a commonly utilized agent for age‐related corrections with minimal adverse effects. Following injection, PLLA undergoes biodegradation, stimulating fibroblasts to produce new collagen, thereby enhancing cosmetic outcomes. Nonetheless, PLLA injections are not devoid of risks, with documented short‐term adverse reactions including pain, swelling, and discoloration, typically resolving within a few weeks.^1^, ^3^, ^5^, ^9^ Late‐onset complications such as granulomas have been reported, necessitating diverse treatment approaches including intralesional steroids. We present a case of late‐onset granuloma in a Korean patient, stressing the significance of informed patient consent and vigilant post‐injection monitoring for potential complications. The rapid improvement observed with intralesional triamcinolone injection highlights the importance of promptly identifying and managing adverse events associated with PLLA filler injections. This paper reports the oldest late‐onset granuloma following PLLA injection, as observed through ultrasound examination, among previously documented cases.

## CONFLICT OF INTEREST STATEMENT

I acknowledge that I have considered the conflict of interest statement included in the “Author Guidelines.” I hereby certify that, to the best of my knowledge, that no aspect of my current personal or professional situation might reasonably be expected to significantly affect my views on the subject I am presenting.

## Supporting information

Supplemental information of ultrasonographic observation of granuloma.

## Data Availability

The data that support the findings of this study are available from the corresponding author upon reasonable request.

## References

[1] Lowe NJ , Maxwell CA , Patnaik R . Adverse reactions to dermal fillers: review. Dermatol Surg. 2005;31(11 Pt 2):1616‐1625 16416647

[2] Olivier Masveyraud F . Facial rejuvenation using L‐polylactic acid: about 298 successive cases. Ann Chir Plast Esthet. 2011;56(2):120‐127. doi:10.1016/j.anplas.2010.09.011 20965636

[3] Lam SM , Azizzadeh B , Graivier M . Injectable poly‐L‐lactic acid (Sculptra): technical considerations in soft‐tissue contouring. Plast Reconstr Surg. 2006;118(3 Suppl):55S‐63S. doi:10.1097/01.prs.0000234612.20611.5a 16936545

[4] Stewart DB , Morganroth GS , Mooney MA , Cohen J , Levin PS , Gladstone HB . Management of visible granulomas following periorbital injection of poly‐L‐lactic Acid. Ophthalmic Plast Reconstr Surg. 2007;23(4):298‐301. doi:10.1097/IOP 17667102

[5] Narins RS . Minimizing adverse events associated with poly‐L‐lactic acid injection. Dermatol Surg. 2008;34(Suppl 1):S100‐S104. doi:10.1111/j.1524-4725.2008.34250.x 18547172

[6] Storer M , Euwer R , Calame A , Kourosh AS . Late‐onset granuloma formation after poly‐l‐lactic acid injection. JAAD Case Rep. 2016;2(1):54‐56. 10.1016/j.jdcr.2015.11.017 27051828 PMC4809477

[7] Lee JJ , Wang YP , Wu YH , Chang JYF . Poly‐l‐lactic acid injection‐induced delayed‐onset foreign body granuloma. J Formos Med Assoc. 2017;116(5):402‐403. doi:10.1016/j.jfma.2016.11.011 28007465

[8] Yi KH , Kim S‐B , Kim H‐J . Ultrasonographic observations of the paradoxical mentalis bulging in regard to botulinum neurotoxin injection for mentalis muscle. Skin Res Technol. 2023;29(11):e13517. doi:10.1111/srt.13517 38009025 PMC10616537

[9] Oh S , Seo SB , Kim G , Batsukh S , Son KH , Byun K . Poly‐D, L‐lactic acid stimulates angiogenesis and collagen synthesis in aged animal skin. Int J Mol Sci. 2023;24(9)7986. doi:10.3390/ijms24097986 37175693 PMC10178436

[10] Oh S , Seo SB , Kim G , et al. Poly‐D,L‐lactic acid filler increases extracellular matrix by modulating macrophages and adipose‐derived stem cells in aged animal skin. Antioxidants (Basel). 2023;12(6)1204. doi:10.3390/antiox12061204 37371934 PMC10294940

